# Trajectories and Depressive Symptoms During the Perinatal Period: A Longitudinal Population-Based Study in China

**DOI:** 10.3389/fpsyt.2022.762719

**Published:** 2022-03-31

**Authors:** Ciqing Bao, Dongzhen Jin, Shiyu Sun, Ling Xu, Chaoyue Wang, Weina Tang, Wenmiao Zhang, Yin Bao, Dongwu Xu, Siyao Zhou, Xin Yu, Ke Zhao

**Affiliations:** ^1^Wenzhou Seventh People's Hospital, Wenzhou, China; ^2^Department of Preventive Medicine, School of Public Health and Management, Wenzhou Medical University, Wenzhou, China; ^3^School of Mental Health, Wenzhou Medical University, Wenzhou, China; ^4^Shaoxing 7th People's Hospital, Shaoxing, China; ^5^Department of Obstetrics, First Affiliated Hospital of Wenzhou Medical University, Wenzhou, China

**Keywords:** depression, social support, perinatal, sleep quality trajectory, parenting experience

## Abstract

Most women in the perinatal period face sleep issues, which can affect their mental health. Only a few studies have focused on sleep trajectories and depressive symptoms of women during the perinatal period in China. This study aims to explore the development trajectory of sleep quality by classifying pregnant women according to the changes in their sleep quality during pregnancy and postpartum and investigate the correlation between different sleep quality trajectory groups and depressive symptoms. The Pittsburgh Sleep Quality Index (PSQI) was used to assess the sleep quality, and the Edinburgh Postnatal Depression Scale (EPDS) was used to assess the symptoms of depression. Participants (*n* = 412) completed the assessment of sleep quality, depressive symptoms, and some sociodemographic and obstetric data at 36 weeks of gestation, 1 week after delivery, and 6 weeks after delivery. The group-based trajectory model (GBTM) was used to complete the trajectory classification, and logistic regression was used to analyze the predictive factors of postpartum depressive symptoms. Four different sleep quality trajectories were determined: “stable-good,” “worsening,” “improving,” and “stable-poor” groups. The results demonstrate that poor sleep trajectories, social support and parenting experience during the perinatal period are related to postpartum depression. Screening for prenatal sleep problems is crucial for identifying the onset of perinatal depressive symptoms.

## Introduction

Perinatal depression is characterized by the occurrence of depressive episodes during pregnancy and the perinatal period with its international prevalence rates ranging from 8 to 36% (1). This disorder is related to many adverse outcomes for women's maternal and physical health. It can interfere with normal maternal–infant relationships and may adversely affect child development (2). However, the specific cause of depression is yet to be determined, especially during the perinatal period. Many risk factors, such as stressful life events in the past 12 months, lack of family support during pregnancy, and low socioeconomic status, among others affect the occurrence of perinatal depression (3, 4).

Countless changes are introduced during the perinatal period. An important but often overlooked change is the increased risk of sleep disorders. In total, 66–94% of pregnant women reported sleep disturbances, resulting mainly from significant changes in their physiology and psychology during pregnancy (5, 6). Most studies demonstrate that decline in sleep quality and night awakenings occur during the third trimester (7–9) and after delivery (10, 11). Sleep gradually worsens with the progression of pregnancy (11–13), usually lasting until the postpartum period and reaching its peak within 1 month after delivery (14). Previous studies show that sleep disturbance may be a risk factor for postpartum depression (13, 15). Dørheim et al. conducted a study on 2,816 women in the 32nd week of pregnancy and found that depressive symptoms were closely related to insomnia in the third trimester of pregnancy (16). Likewise, Skouteris et al. supports the view that sleep disturbance is related to an increase in depressive symptoms during pregnancy (17). Besides this, Park et al. also conducted objective and subjective assessments of sleep quality from the third trimester to the postpartum period. They used the General Sleep Disturbance Scale to evaluate subjective sleep quality and the Edinburgh Postnatal Depression Scale to evaluate depression and found that sleep quality was highly correlated with depressive symptoms (18). The hypothalamic–pituitary–adrenal (HPA) axis may explain the potential biological mechanism between sleep quality and depression. The HPA axis is a physiological system that can be activated by stress, which leads to the release of corticotropin (ACTH) from the pituitary gland and cortisol from the adrenal glands. In general, cortisol levels have a significant circadian rhythm in humans with higher levels in the morning and lower levels in the evening (19). People with poor sleep quality can activate the responsiveness of the HPA axis to physical and psychosocial stressors. The HPA axis is also related to depression (20). Researchers have found significantly increased plasma corticosterone levels in the classic depression model of rodents (21). Thus, an abnormal HPA axis may participate in the manifestation of sleep disturbance and depression. Moreover, psychosocial factors can also impart variability in this association between sleep disturbance and depression. The postpartum period is a special period for new mothers when they may not be able to adapt to the role change for a while. They may find it difficult to take care of the newborn. Difficult infant temperaments are recognized as important stressors for mothers throughout the postpartum period (22), and these can impair the sleep quality experienced by mothers. Mothers with insufficient sleep may feel more pessimistic, thus underestimating their social support status (23). Furthermore, sleep quality affects women's ability to cope with motherhood and future expectations, both practically and emotionally (24, 25). Goyal et al. found that prolonged nighttime wakefulness (>2 h) is associated with more severe depressive symptoms (26).

When investigating sleep quality in the perinatal period, many studies focus on changes in the average sleep quality over time in the perinatal period (27, 28), ignoring the fact that not all pregnant women experience the same pattern of sleep changes during the perinatal period. Although sleep disorders are correlated with the appearance of new depressive symptoms during pregnancy (17), it seems inappropriate to use a fixed sleep pattern to explain perinatal depression due to individual differences. Therefore, it is of great significance to study the sleep quality trajectory, which is used to distinguish different subgroups of sleep quality. Some previous studies explore the correlation between sleep quality trajectories and postpartum depression. Because of differences in grouping standards and statistical methods, the trajectories of sleep vary in different previous studies. For example, Tomfohr et al. chose four-time nodes to measure sleep quality and found that subgroups having a considerable decline in sleep quality from early to late pregnancy as well as poor sleep quality throughout pregnancy were more likely to experience depression in the postpartum period (29). This study ignores the time note of 6 weeks following delivery because it is a time when postpartum depression was of high incidence (30). Wang et al. chose more time nodes to evaluate sleep quality and found that poor sleep quality increased mood disorders at 36 months postpartum (31), but it ignored some psychosocial stressors. Because psychosocial stressors are risk factors that can affect sleep and perinatal depression, they should not be ignored when studying sleep quality trajectories and depressive symptoms.

In Asia, there are currently just a few studies on monitoring sleep trajectories during the perinatal period. Exploring sleep trajectories will help in better understanding the relationship between sleep and perinatal depression and provide timely and targeted interventions for more personalized treatment. Therefore, the objectives of this study are to (1) investigate the sleep trajectory of the Asian population during the perinatal period, (2) explore the differences in social and psychological factors among the trajectory groups, and (3) study the relationship between sleep trajectory and maternal depressive symptoms. Women with the greatest increase in sleep problems were assumed to be the most likely to experience high-level depressive symptoms during the postpartum period.

## Methods

This study is a longitudinal investigation of all pregnant women who underwent an obstetric examination and will give birth in the First Affiliated Hospital of Wenzhou Medical University. Participants will be enrolled in the third trimester of pregnancy, and the investigator will only further screen women for eligibility after obtaining oral consent. We employed a structured neuropsychiatric interview, the Mini International Neuropsychiatric Interview, to evaluate the presence of psychiatric disorders (32). The inclusion criteria are (1) 18–40 years old, (2) 28 weeks of pregnancy or more, (3) regular check-ups in the research hospital, (4) elementary school and higher education, (5) no sleep problems before pregnancy, and (6) voluntarily signed informed consent. Women with severe pregnancy complications or any mental/cognitive problems, including pregnant women with a history of prenatal depression, other mental illnesses, and intellectual disability, that would prevent them from completing the survey were excluded from this study.

### Procedure

The pregnant women who agreed to participate in the study underwent obstetric examination and data collection at three time points: T1 (gestational week 36), T2 (within 1 week postpartum) and T3 (6 weeks postpartum). Two psychiatrists trained three graduate students who major in psychiatry before the research. The graduate students were taught to fully comprehend the specific meaning of each item in each scale and know how to respond to the issues raised by participants and guide them in completing the whole experiment. Once the participant fills out the informed consent form, the researchers will help the participant complete the survey. This study was approved by the Ethics Committee of Wenzhou Medical University.

This study is part of a larger longitudinal study of maternal mental health being conducted at the First Affiliated Hospital of Wenzhou Medical University. From December 2017 to January 2019, a total of 988 people completed enrollment with 667 completing the first follow-up and 412 people completing the second follow-up, hence making the final sample of this study to 412 women. There was a 42% completion rate among the 988 initially enrolled participants.

### Measures

Participants completed the Pittsburgh Sleep Quality Index (PSQI) and Edinburgh Postpartum Depression Scale (EPDS) at three time points (T1, T2, and T3). Furthermore, a self-edited demographic questionnaire survey was conducted at T1, and the Social Support Rating Scale (SSRS) and postpartum data update were completed at T2. The initial follow-up was conducted in the ward. The researchers contacted the participants before the second follow-up to confirm the review date, and the participants completed the second follow-up in the obstetric clinic.

### Self-Edited Census Form

Under the supervision of a well-trained evaluator, pregnant women filled out a social demographic survey, including age, gestational age, BMI, years of education, race, place of residence, marital status, family monthly income, currently smoking and drinking, long-term exercise habits, and the number of existing children. In addition, following delivery, updated obstetric data, such as planned pregnancy, postpartum complications, delivery methods, etc., were also incorporated in the survey.

### Evaluation of Sleep Quality

The PSQI (33) was used to evaluate the preceding month's sleep quality, and it has a maximum total score of 21 points. In total, 19 individual items generate seven component scores (range 0–3 with higher scores indicating worse sleep): subjective sleep quality, sleep latency, sleep duration, habitual sleep efficiency, sleep disturbances, use of sleeping medication, and daytime dysfunction. This scale has good internal consistency, test–retest reliability, and validity (34). A score of >5 indicates clinically significant sleep disruption (35).

### Evaluation of Depressive Symptoms

The EPDS (36) is a frequently used depression assessment scale in Western countries. It is believed to be effective during pregnancy and postpartum in multiple countries (37–40). The EPDS investigates 10 items: mood, pleasure, guilt, anxiety, fear, insomnia, ability to cope, sadness, crying, and self-injury. Each item is divided into 0–3 points in accordance with the severity of the relevant symptom, and the total score ranges from 0 to 30 points. The scale has good reliability and validity among populations in Mainland China (41). A total score of >9 was classified as depression in this experiment (42).

### Social Support Assessment

The SSRS is used to evaluate the social support of pregnant women. The Chinese version of SSRS was first developed by Xiao in 1986 (43). It comprises objective support, subjective support, and availability. The total score ranges from 12 to 66 points. The higher the score, the higher the level of social support. The scale has been developed and widely used in China and has high reliability and validity.

### Statistical Analysis

GBTM (44) was used to identify sleep quality trajectories from late pregnancy to 6 weeks postpartum. Such models use a semiparametric approach to identify a set of curves (the trajectories) that capture the main features of the data with each curve representing a different trajectory group. Unlike traditional growth curve models that assume a single average population growth trajectory, GBMT does not assume a unique potential growth trajectory and considers within-population heterogeneity. The polynomial equation modeling was used to model the relationship between time (perinatal) and outcome (sleep quality) to best define the trajectory. To obtain information that maximizes relevant heterogeneity while maintaining parsimony, the number of subgroups was selected by the following criteria: (1) the absolute value of Bayesian information criterion (BIC) was minimal, (2) the number of people in each group was not <5% of the sample, (3) it was clinically interpretable, and (4) the increased trajectory showed essential features in the data.

Continuous variables were expressed as mean ± standard deviation (SD), whereas categorical data were described by numbers (percentages). The normally distributed data were analyzed by one-way analysis of variance, whereas non-parametric data are analyzed by Kruskal–Wallis test statistics. Categorical data were evaluated by the chi-square test. Multiple comparisons among the four groups were performed after the significance test to better understand the difference between the baseline variables. After establishing the meaningfulness of the Kruskal–Wallis test, the least significant difference (LSD) method is used for pairwise comparison. After making corrections, the chi-square segmentation method is used for pairwise comparison after establishing the meaningfulness of the chi-square test. Finally, multivariate logistic regression was used to determine the independent effects of the sleep quality trajectory group on depression. All results were analyzed using IBM SPSS Statistics 22.0 and Stata 14.1, and *p* < 0.05 on both sides was considered statistically significant.

## Results

### Characteristics of the Study Population

The study included 988 pregnant women, of whom 412 completed all assessments. Those who completed all assessments were regarded as responders (*n* = 412), and those who did not complete all assessments were regarded as non-responders (*n* = 576). Table 1 shows that there is no significant difference in T1 sleep status and depression between pregnant women who withdrew from follow-up during the T2 and T3 time periods and those who completed all three evaluations (both *p*s > 0.05).

**Table 1:** Socio-demographics and clinical characteristics between the groups of responders and non-responders.

**Variables**	**Responders (***N*** = 412)**	**Non-responders (***N*** = 576)**	***P***
Age, years	28.5 (4.09)	29.1 (4.26)	0.021^*^
BMI, kg/m^2^	25.3 (2.89)	25.3 (3.05)	0.763
Education, years	13.5 (2.59)	13.2 (2.97)	0.493
Gestational week	36.10 (34.50, 37.20)	36.00 (34.50, 37.10)	0.484
Currently married, %	409 (99.3)	565 (98.1)	0.121
Currently drinking, %	18 (4.4)	32 (5.6)	0.401
Currently smoking, %	3 (0.7)	12 (2.1)	0.086
Residence, %			0.108
Town	141 (34.2)	226 (39.2)	
Countryside	271 (65.8)	350 (60.8)	
Monthly household income, %			0.001^**^
<5,000 (RMB)	81 (19.7)	143 (24.8)	
5,000–10,000 (RMB)	201 (48.8)	213 (37.0)	
>10,000 (RMB)	130 (31.6)	220 (38.2)	
Long-term exercise habits			0.199
No	355 (86.2)	479 (83.2)	
Yes	57 (13.8)	97 (16.8)	
Have children			0.060
0	231 (56.1)	297 (51.6)	
1	171 (41.5)	213 (47.4)	
≥2	10 (2.4)	6 (1.0)	
EPDS (T1)	7.6 (3.74)	7.9 (3.96)	0.204
PSQI (T1)	6.4 (3.28)	6.6 (3.42)	0.222

*EPDS, Edinburgh Postpartum Depression Scale; PSQI, Pittsburgh Sleep Quality Index*.

* *p < 0.05*,

** *p < 0.01*,

*** *p < 0.001*.

### Identification of Perinatal Sleep Quality Trajectories

The main objective of the trajectory analysis model is to determine the appropriate number of subgroups and the shape of each subgroup. To determine the best one, the model was first determined based on only one trajectory. It was found that obtaining the trajectory twice was more appropriate (BIC = −3,258.14). Then, models of two (BIC = −3,163.27), three (BIC = −3,147.67), four (BIC = −3,144.12), and five trajectories (BIC = −3,139.26) were estimated. Although the BIC is the smallest in a model of five trajectories, one of the groups has nine people (2.18% < 5%). Finally, four groups are selected as the best fit model.

### Characteristics of the Trajectory Groups

**Figure 1:**
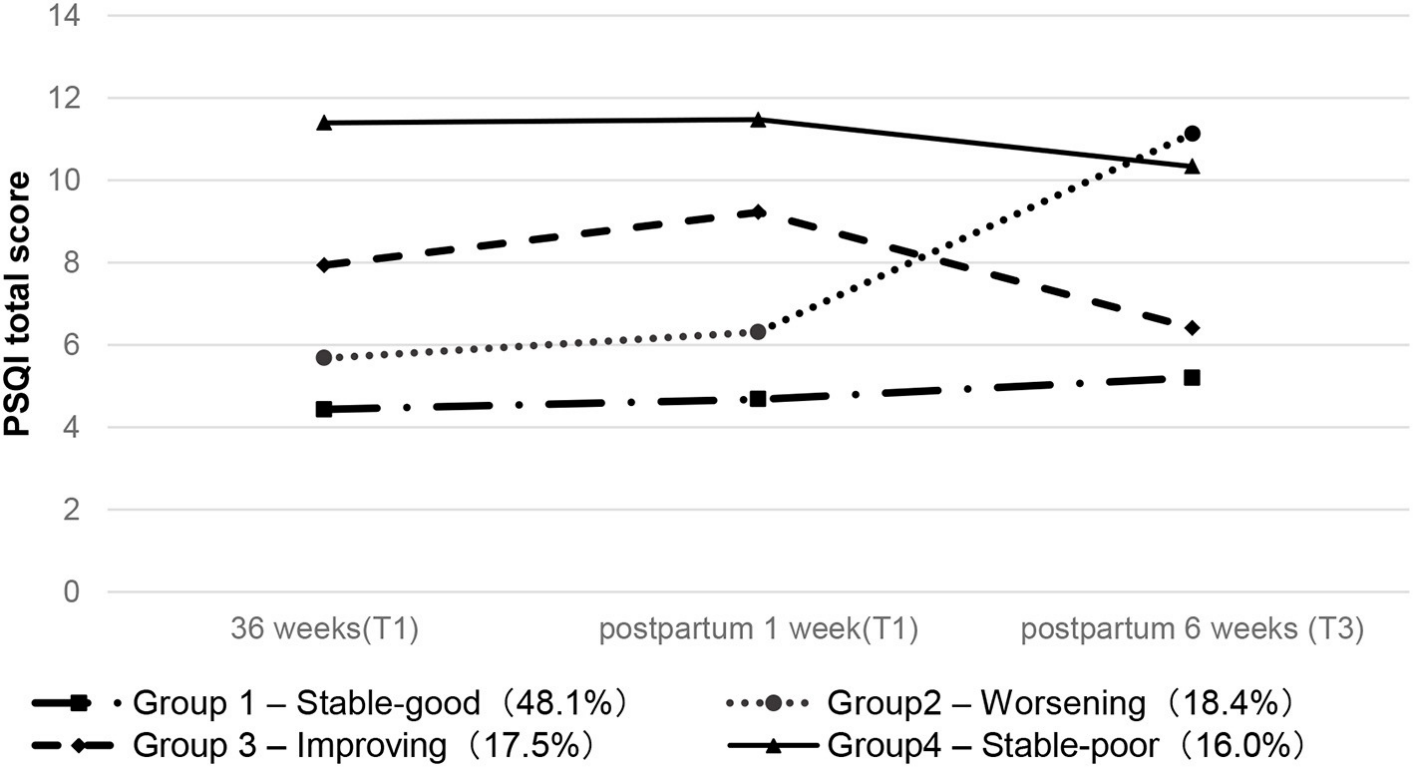
Latent trajectories of four groups about sleep quality.

Figure 1 shows the identified four trajectories to reflect the changes in sleep quality. The first group is the “stable-good” group. The sleep quality of patients in this group fluctuates a little, and the PSQI scores are all below six points. The second group was defined as the “worsening” group. The PSQI value of women in this group continued to rise, reaching a peak at 6 weeks postpartum. The third group is the “improving” group. Women in this group suffer from poor sleep during the third trimester, which continues to deteriorate, but the score drops 1 week after delivery and is close to normal at 6 weeks after delivery. The fourth group is defined as the “stable-poor” group, and the PSQI score has always been at a high level.

Table 2 and Supplementary Table 1 show the differences in baseline characteristics of the sleep trajectory groups. Other demographic data did not differ across sleep trajectory groups (*P* > 0.05) except for years of education (*P* = 0.003). After pairwise comparison and correction (LSD method), the number of years of education in the “stable-poor” group was lower than in the “stable-good” group (*P* = 0.006). Compared with the “stable-good” group, the EPDS scores of the other three trajectory groups were higher with statistically significant differences (*P* < 0.001), but there was no difference among the other three trajectory groups after pairwise comparison.

**Table 2:** Differences in baseline characteristics among sleep trajectory groups.

**Variable**	**Group 1 ***N*** = 198**	**Group 2 ***N*** = 76**	**Group 3 ***N*** = 72**	**Group 4 ***N*** = 66**	***P***
Age, years	28.04 (3.96)	28.62 (3.99)	29.31 (4.13)	28.85 (4.46)	0.114
BMI, kg/m^2^	24.96 (3.18)	25.49 (3.08)	25.60 (3.26)	25.09 (2.98)	0.738
Education, years	13.84 (2.50)	13.18 (2.46)	13.08 (3.21)	12.85 (2.45)	0.003^**^
Currently married, %	197 (99.5)	75 (98.7)	72 (100.0)	65 (98.5)	0.587
Currently drinking, %	6 (3.0)	2 (2.6)	4 (5.6)	6 (9.1)	0.160
Currently smoking, %	1 (0.5)	1 (1.3)	1 (1.4)	0 (0.0)	0.529
Residence, %					0.877
Town	66 (33.3)	27 (35.5)	23 (31.9)	25 (37.9)	
Countryside	132 (66.7)	49 (64.5)	49 (68.1)	41 (62.1)	
Monthly household income, %					0.426
<5,000 (RMB)	39 (19.7)	15 (19.7)	15 (20.8)	13 (19.7)	
5,000–10,000 (RMB)	97 (49.0)	42 (55.3)	36 (50.0)	25 (37.9)	
>10,000 (RMB)	62 (31.3)	19 (25.0)	21 (29.2)	28 (42.4)	
Long-term exercise habits					0.717
No	172 (86.9)	66 (86.8)	59 (81.9)	58 (87.9)	
Yes	26 (13.1)	10 (13.2)	13 (18.1)	8 (12.1)	
Have children now					0.739
0	109 (35.1)	45 (59.2)	45 (62.5)	31 (47.0)	
1	84 (42.4)	28 (36.8)	27 (37.5)	33 (50.0)	
≥2	5 (2.5)	3 (3.9)	1 (0.0)	2 (3.0)	
EPDS (T1)	6.22 (3.01)	7.84 (3.67)	9.07 (3.45)	9.85 (4.40)	<0.001^***^
PSQI (T1)	4.34 (1.79)	5.63 (1.90)	8.13 (1.92)	11.39 (2.76)	<0.001^***^

*Group 1, the stable-good group; Group 2, the worsening group; Group 3, the improving group; Group 4, the stable-poor group. EPDS, Edinburgh Postpartum Depression Scale; PSQI, Pittsburgh Sleep Quality Index*.

* *p < 0.05*,

** *p < 0.01*,

*** *p < 0.001*.

Table 3 shows the updated obstetric data following delivery. SSRS, EEPDS, planned pregnancy, delivery method, feeding method, and expectations of the baby's gender were statistically different among the four sleep trajectory groups (all *p* < 0.05). After pairwise comparison (Supplementary Table 2), the social support scores of the “improving” and the “stable-poor” groups were lower than that of the “stable-good” group (*p* < 0.05). The degree of compliance with the baby's gender expectations was lower in the “worsening” group (*p* < 0.0071). The incidence of cesarean section was higher in the “improving” group (*p* < 0.0071).

**Table 3:** Comparison among the sleep trajectory groups after delivery.

**Variable**	**Group 1 ***N*** = 198**	**Group 2 ***N*** = 76**	**Group 3 ***N*** = 72**	**Group 4 ***N*** = 66**	***P***
EPDS (T2)	5.45 (2.85)	7.17 (3.49)	7.32 (3.43)	8.30 (4.55)	<0.001^***^
SSRS (T2)	30.52 (3.79)	29.00 (4.41)	28.38 (4.18)	28.02 (4.80)	<0.001^***^
Planned pregnancy					0.038^*^
Yes	133 (67.2)	40 (52.6)	37 (51.4)	42 (63.6)	
No	65 (32.8)	36 (47.4)	35 (48.6)	24 (36.4)	
Postpartum complications					0.489
No	184 (92.9)	71 (93.4)	63 (87.5)	60 (90.9)	
Yes	14 (7.1)	5 (6.6)	9 (12.5)	6 (9.1)	
Full-term birth					0.840
No	3 (1.5)	2 (2.6)	2 (2.8)	2 (3.0)	
Yes	195 (98.5)	74 (97.4)	70 (97.2)	64 (97.0)	
Delivery method					0.011^*^
Vaginal delivery	156 (78.8)	52 (68.4)	43 (59.7)	44 (66.7)	
Cesarean section	42 (21.2)	24 (31.6)	29 (40.3)	22 (33.3)	
Dystocia					0.597
No	192 (97.0)	73 (96.1)	68 (94.4)	65 (98.5)	
Yes	6 (3.0)	3 (3.9)	4 (5.6)	1 (01.5)	
Fetal sex habits					0.172
Boy	107 (54.0)	39 (51.3)	37 (52.1)	26 (39.4)	
Girl	91 (46.0)	36 (47.4)	34 (47.9)	40 (60.6)	
Boy and girl	0 (0.0)	1 (1.3)	0 (0.0)	0 (0.0)	
Gender expectations					0.020^*^
Satisfaction	187 (94.4)	63 (82.9)	63 (87.5)	57 (86.4)	
Dissatisfied	11 (5.6)	13 (17.1)	9 (12.5)	9 (13.6)	
Feeding method					0.823
Breastfeeding	184 (92.9)	72 (94.7)	66 (91.7)	60 (90.9)	
Non-breastfeeding	14 (7.1)	4 (5.3)	6 (8.3)	6 (9.1)	
Parenting experience					0.505
Yes	92 (46.5)	32 (42.1)	30 (41.7)	35 (53.0)	
No	106 (53.5)	44 (57.9)	42 (58.3)	31 (47.0)	

*Group 1, the stable-good group; Group 2, the worsening group; Group 3, the improving group; Group 4, the stable-poor group; EPDS, Edinburgh Postpartum Depression Scale; SSRS, The Social Support Rating Scale*.

* *p < 0.05*,

** *p < 0.01*,

*** *p < 0.001*.

### Predictors of Membership in Postpartum Depression

Finally, multivariate logistic regression was used to check whether sleep trajectories are associated with an increased risk of postpartum depression symptoms (Table 4). At the same time, variables including age, BMI, years of education, monthly family income, currently drinking, parenting experience, and social support (SSRS) that may cause postpartum depression were added into the model. The results show that the “stable-good” group was the reference group in the comparison of the sleep track group. Compared with the “stable-good” group, there was no significant difference in the “improving” group. The “worsening” and “stable-poor” groups had higher incidence of postpartum depression symptoms (AOR is 2.56 and 3.57, respectively). For every one-point increase in the social support scale score, the probability of postpartum depression decreased by 12% (*P* < 0.001). Besides this, pregnant women with childcare experience had a lower risk of depression than those without childcare experience (AOR = 0.49).

**Table 4:** Multivariate logistic regression for risks of postpartum depression symptoms.

**Variables**	**Unadjusted model**		**Adjusted model**	
	**OR (95%CI)**	***P*** **-value**	**AOR (95%CI)**	***P-*** **value**
**Group**				
Stable-good	1 (Reference)		1 (Reference)	
Worsening	2.88 (1.40–5.95)	0.004^**^	2.56 (1.20–5.48)	0.015^*^
Improving	2.00 (0.91–4.39)	0.084	1.54 (0.67–3.54)	0.311
Stable-poor	4.35 (2.13–8.88)	<0.001^***^	3.57 (1.64–7.76)	0.001^**^
SSRS	0.86 (0.81–0.92)	<0.001^***^	0.88 (0.82–0.94)	<0.001^***^
**Parenting experience**				
No	1 (Reference)		1 (Reference)	
Yes	0.52 (0.30–0.91)	0.021^*^	0.49 (0.25–0.96)	0.038^*^

*AOR, adjusted odds ratio; CI, confidence interval; OR, odds ratio; SSRS, Social Support Rating Scale; Group, perinatal sleep quality trajectory groups*.

*Adjusted for age, BMI, education, currently drinking, Monthly household income, SSRS, parenting experience*.

* *p < 0.05*,

** *p < 0.01*,

*** *p < 0.001*.

## Discussion

This longitudinal study of sleep quality trajectory during the perinatal period among Chinese women demonstrated four distinct trajectories based on the GBTM. From the third trimester to 6 weeks postpartum, four different sleep trajectories were found: “stable-good” (group 1), “worsening” (group 2), “improving” (group 3), and “stable-poor” (group 4). Importantly, trajectories of sleep quality were also found to be associated with depressive symptoms. Even after controlling for relevant demographic factors and childbirth-related covariates, the track group with the worst sleep quality in the third trimester was most likely to have high-level depressive symptoms in the postpartum period. Besides this, the time points of detection we chose were reasonable, and this study highlights the role of social and psychological factors among different sleep quality trajectory groups on depressive symptoms in the postpartum period.

Most of the previous studies on sleep quality and perinatal depression are based on cross-sectional sleep quality to predict depression. However, this study found that the trajectory of perinatal sleep is not static, meaning that certain limitations are present in predicting postpartum depression based on sleep quality at a time point. A significant correlation was found between sleep trajectory groups (the “worsening” and “stable-poor” group) and postpartum depression in this study. It is believed that longitudinal sleep trajectories may be a better predictor of postpartum depression. Although there are relatively few studies on the perinatal sleep trajectory group, this study's findings are consistent with the previous ones (29, 31, 45–47). In the past, there was also some longitudinal evidence of an association between pregnant women's sleep quality during pregnancy and postpartum depression (48, 49), but there were fewer studies in the perinatal trajectory group and poor sleep quality/shortened sleep time/insomnia during the perinatal period. Women with poor sleep trajectories are more likely to suffer from depression (29, 31, 45, 50), and this is also reflected in the women with cesarean section (47).

At present, the relationship between sleep and perinatal depression is not very clear. As we all know, the perinatal period is a special stage marked by great physical and mental changes. Therefore, it is believed that the relationship between them may be attributed to a combination of social, psychological, and physiological factors. Most previous sociodemographic studies find that multiple factors, such as age, income, education, and social support, were involved in the relationship between sleep and postpartum depression (51). In addition, the related neurotransmitters (estrogen, progesterone) that regulate sleep quality were also involved in the regulation of emotions. Therefore, the sharp drop in estrogen and progesterone levels after childbirth aggravate sleep disturbances in women, leading to postpartum depression (52, 53). Sustained sleep time shortening/sleep deprivation in postpartum women was related to the increase of systemic inflammation. Severe systemic inflammation itself was associated with postpartum depression (54).

This study's results show that, except for years of education and prenatal EPDS scores, there were no significant differences among the sleep trajectory groups in other sociodemographic factors during the baseline period. The number of years of education in the “stable-good” group was found to be significantly higher than that of the “stable-poor” group. It is well-documented that people with low years of education have more frequent sleep problems (55, 56). The “stable-good” group were found to have the lowest depressive symptoms at baseline, the “stable-poor” group had the highest level of depression, and the other two groups were in the middle. This result is consistent with previous results stating that the trajectory of poor sleep quality was associated with higher depressive symptoms at baseline (29, 31).

In terms of postpartum, there are also significant differences among the trajectory groups. Among them, the “stable-good” group has the best social support, whereas the “stable-poor” group has the worst social support, meaning that pregnant women with low social support are more likely to face sleep problems (29). At the same time, this research also concludes that social support is negatively correlated with postpartum depression, further confirming that social support may be a sociological factor related to perinatal sleep and postpartum depression. Compared with the “stable-good” group, the “worsening” group has lower expectations for the baby's gender. This trajectory group is one of the important factors for the continuous worsening of sleep of pregnant and lying-in women. Finally, the incidence of cesarean section in the “improving” group is higher, and it is believed that its possible mechanism is that there are more cesarean sections in this trajectory group within 1 week after delivery, and wound pain has a certain impact on sleep. As the wound improves, the impact of cesarean section decreases and sleep improves gradually. It is worth mentioning that there is a negative correlation between parenting experience and postpartum depression, which may be related to the important life event of childbirth. The transition from a woman to being a “mother” is a complex process that may lead to psychological disorders or psychopathological conditions, such as postpartum depression. Previous studies show that the risk of postpartum depression for primiparous women is greater than multiparas women (57, 58), which indicates that women with parenting experience may have a lower risk of postpartum depression than those without parenting experience. Providing parenting support for new mothers may help reduce their suffering, and some studies also support our view (59, 60). In the future, parenting-related education for primipara should be valued, which may reduce the incidence of postpartum depression.

This study has some limitations. First, it has a small sample size for all assessments and a higher rate of loss to follow-up. Second, the assessment of sleep quality comes from a subjective scale. Although PSQI is an effective measurement method, objective (such as activity recording) sleep measurement methods are more important. In addition, the measurement and evaluation points are too few, and future research should focus on the entire perinatal period and the period even before pregnancy. It should also be noted that significant differences are present between responders (completed three assessments) and non-responders. Respondents were younger and had a lower family monthly income ratio, but there was no statistically significant difference in the evaluation scores of prenatal sleep and childbirth depression symptoms between the two groups. Finally, future efforts will focus on expanding the sample size and reducing the loss to follow-up rate.

## Conclusion

This study clarified the four trajectories of perinatal sleep and the correlation between sleep trajectories and postpartum depression. The results demonstrate that the “stable-poor” sleep trajectory group had a higher incidence of postpartum depression. Higher social support and parenting experience are protective factors for postpartum depression. As an extension of this research, it is possible to detect perinatal sleep disturbances as early as possible. In future studies, multiple sleep assessments during the perinatal period can be used as a screening and early intervention tool for stopping the occurrence of postpartum depression and adverse pregnancy outcomes.

## Data Availability Statement

The raw data supporting the conclusions of this article will be made available by the authors, without undue reservation.

## Ethics Statement

The studies involving human participants were reviewed and approved by the Medical Ethics Committee of the Wenzhou Medical University. The patients/participants provided their written informed consent to participate in this study.

## Author Contributions

KZ, XY, and SZ conceived and designed the study. CB developed it in discussion with SS, LX, WT, and CW. DJ, CW, WZ, YB, and DX were involved in the acquisition and analysis of the data. CB wrote the first draft of the article. All authors contributed to critically revising the paper, participated in the interpretation of the data, read, and approved the final manuscript.

## Funding

This work was supported by the Zhejiang Provincial Natural Science Foundation (LY19H090015 and LY22H090022) and the Wenzhou Science and Technology Bureau project (Y20210763 and S20180012). The preparation of material scales is part of the fund's funding.

## Conflict of Interest

The authors declare that the research was conducted in the absence of any commercial or financial relationships that could be construed as a potential conflict of interest.

## Publisher's Note

All claims expressed in this article are solely those of the authors and do not necessarily represent those of their affiliated organizations, or those of the publisher, the editors and the reviewers. Any product that may be evaluated in this article, or claim that may be made by its manufacturer, is not guaranteed or endorsed by the publisher.
