# Supplementary material for: Trajectories and Depressive Symptoms During the Perinatal Period: A Longitudinal Population-Based Study in China
Source: Front Psychiatry. 2022 Mar 31;13:762719. doi: 10.3389/fpsyt.2022.762719 (PMC9009256; doi:10.3389/fpsyt.2022.762719)
Supplement: Supplementary file 1 [file Data_Sheet_1.pdf]

Supplementary Table 1 Comparison among the sleep trajectory groups in baseline.

| Variables | 1v2 | 1v3 | 1v4 | 2v3 | 2v4 | 3v4 |
|---|---|---|---|---|---|---|
| Education, year | 0.134 | 0.474 | 0.006** | 1.000 | 1.000 | 1.000 |
| EPDS（T1） | 0.008** | ＜0.001*** | ＜0.001*** | 0.269 | 0.057 | 1.000 |
| PSQI（T1） | ＜0.001*** | ＜0.001*** | ＜0.001*** | ＜0.001*** | ＜0.001*** | 0.004** |

Note: 1 Group1 the stable-good group; 2 Group2 the worsening group; 3 Group3 the improving group;4 Group4 the stable-poor group. EPDS Edinburgh Postpartum Depression Scale; PSQI Pittsburgh Sleep Quality Index; * p<0.05, ** p<0.01, *** p<0.001.

Supplementary Table 2 Comparison among the sleep trajectory groups after delivery.

| Variables | 1v2 | 1v3 | 1v4 | 2v3 | 2v4 | 3v4 |
|---|---|---|---|---|---|---|
| EPDS（T2） | 0.001** | 0.001** | ＜0.001*** | 1.000 | 1.000 | 1.000 |
| SSRS（T2） | 0.058 | 0.002** | 0.001** | 1.000 | 1.000 | 1.000 |
| [a] Planned pregnancy | 0.026 | 0.018 | 0.599 | 0.880 | 0.185 | 0.146 |
| [a] Delivery method | 0.072 | 0.002[#] | 0.047 | 0.270 | 0.824 | 0.399 |
| [a]Gender expectations | 0.002[#] | 0.054 | 0.031 | 0.431 | 0.569 | 0.843 |

Note: 1 Group1 the stable-good group; 2 Group2 the worsening group; 3 Group3 the improving group;4 Group4 the stable-poor group. EPDS Edinburgh Postpartum Depression Scale; SSRS The Social Support Rating Scale; * p<0.05, ** p<0.01, *** p<0.001.

[a]Pairwise comparison was made by chi-square splitting method, corrected $P_c=P*(4*3/2+1)=P^a$,

[#] $p^a<0.0071$
